# Supplementary material for: Identifying missed opportunities in tuberculosis preventive treatment care cascade: Analysis of programme data from Maharashtra, India
Source: PLOS Glob Public Health. 2026 Jan 16;6(1):e0004630. doi: 10.1371/journal.pgph.0004630 (PMC12810897; doi:10.1371/journal.pgph.0004630)
Supplement: S1 File — (DOCX) [file pgph.0004630.s001.docx]

STROBE Statement—checklist of items that should be included in reports of observational studies

|  | Item No. | Recommendation | Page No. | Relevant text from manuscript |
| --- | --- | --- | --- | --- |
| **Title and abstract** | 1 | (*a*) Indicate the study’s design with a commonly used term in the title or the abstract | Page 1, 3 | Text identifying the study involved analysis of secondary data –  **Title** – “Analysis of programme data from Maharashtra, India”  **Abstract** - “We analysed the state programme data” |
|  |  | (*b*) Provide in the abstract an informative and balanced summary of what was done and what was found | Page 3 | We analysed the state programme data to describe the tuberculosis preventive treatment care cascade for household contacts of all notified people with pulmonary tuberculosis for the year 2023 in Maharashtra.  Contact tracing was done for 84% of the 133,167 notified people with pulmonary tuberculosis. A total of 406,291 household contacts were enlisted out of whom 386,224 (95%) were screened for symptoms of tuberculosis. 185,502 (45%) household contacts were listed as eligible for tuberculosis preventive treatment, of whom 101,325 (55%) were initiated on tuberculosis preventive treatment. While 41,480 (41%) of those initiated on treatment successfully completed it, treatment outcomes were not recorded for around 57,191 (56%) of them. Tuberculosis preventive treatment completion as well as recording of treatment outcomes was lesser for 6H regimen, among contacts of those seeking care from private sector and clinically diagnosed people with tuberculosis. |
| Introduction | | | |  |
| Background/rationale | 2 | Explain the scientific background and rationale for the investigation being reported | Page 6 | Line 85-91 |
| Objectives | 3 | State specific objectives, including any prespecified hypotheses | Page 6 | We therefore planned this study with the objective of describing the TPT care cascade for HHC of all notified PwPTB for the year 2023 in the state of Maharashtra. |
| Methods | | | |  |
| Study design | 4 | Present key elements of study design early in the paper | Page 8 | Line 137-139 |
| Setting | 5 | Describe the setting, locations, and relevant dates, including periods of recruitment, exposure, follow-up, and data collection | Page 7-8 | Settings/ locations described under specific settings (line 105-125)  recruitment, exposure, follow-up, and data collection described under study population and data management and analysis sections (line 141-151) |
| Participants | 6 | (*a*) *Cohort study*—Give the eligibility criteria, and the sources and methods of selection of participants. Describe methods of follow-up  *Case-control study*—Give the eligibility criteria, and the sources and methods of case ascertainment and control selection. Give the rationale for the choice of cases and controls  *Cross-sectional study*—Give the eligibility criteria, and the sources and methods of selection of participants | Page 8 | Line 145-151 |
|  |  | (*b*) *Cohort study*—For matched studies, give matching criteria and number of exposed and unexposed  *Case-control study*—For matched studies, give matching criteria and the number of controls per case |  | NA |
| Variables | 7 | Clearly define all outcomes, exposures, predictors, potential confounders, and effect modifiers. Give diagnostic criteria, if applicable | Page 7 | Eligibility criteria defined in line 107 – 113 and line 141-143  Outcomes defined in data management – line 152-157 |
| Data sources/ measurement | 8* | For each variable of interest, give sources of data and details of methods of assessment (measurement). Describe comparability of assessment methods if there is more than one group | *Page 9* | Data, extracted in MS excel format, from the three registers specifically notification register (for information on index patient characteristics), contact tracing register (for information on identification, evaluation and eligibility of HHC), and TPT register (for TPT regimens, outcomes and characteristics of HHC initiated on TPT) |
| Bias | 9 | Describe any efforts to address potential sources of bias | Page 8 | Since this was a secondary data analysis, the scope of limiting any potential source of bias was minimal. We however, tried to minimize some source of measurement bias. Please note the excerpt from the manuscript – “People with extra pulmonary TB, site not recorded, those notified outside the specified period (Jan-Dec 2023), and the records with total number of HHC >20 or number of HHC (aged ≤ 6 years) >10 were excluded. All the identifiers other than unique patient id were removed prior to performing the analysis.” |
| Study size | 10 | Explain how the study size was arrived at | - | Records for all the available potential beneficiaries were included. Since this was analysis of secondary data, we did not calculate a required sample size |

Continued on next page

| Quantitative variables | 11 | Explain how quantitative variables were handled in the analyses. If applicable, describe which groupings were chosen and why | Page 9 | Categorical variables were summarized as frequency and percentages. Continuous variables were summarized as median (Inter-quartile range, IQR). We also assessed the TPT outcomes by sociodemographic characteristics of the HHC, TPT regimen and clinical characteristics of index PwPTB to understand any differences across the different categories. |
| --- | --- | --- | --- | --- |
| Statistical methods | 12 | (*a*) Describe all statistical methods, including those used to control for confounding | Page 9 | Categorical variables were summarized as frequency and percentages. Continuous variables were summarized as median (Inter-quartile range, IQR).  Since we did not use any statistical test of significance, we also did not attempt to control any confounders. Moreover, this being programme-based data, it contained information on very minimal and essential variables. |
|  |  | (*b*) Describe any methods used to examine subgroups and interactions |  | NA |
|  |  | (*c*) Explain how missing data were addressed | Page 8 | Data from notification and contact tracing registers were merged using the unique patient id. People with extra pulmonary TB, site not recorded, those notified outside the specified period (Jan-Dec 2023), and outliers i.e. the records with total number of HHC >20 or number of HHC (aged ≤ 6 years) >10 were excluded. |
|  |  | (*d*) *Cohort study*—If applicable, explain how loss to follow-up was addressed  *Case-control study*—If applicable, explain how matching of cases and controls was addressed  *Cross-sectional study*—If applicable, describe analytical methods taking account of sampling strategy | Page 9 | Line 155 – “Outcomes labelled as died, lost to follow-up, treatment failed, TPT discontinuation due to toxicity, and not evaluated were categorized as ‘TPT not completed’.”  Loss to follow up is itself an outcome of interest. It is being recorded and reported as such by the programme. The proportion of loss to follow up has been mentioned in figure 2. |
|  |  | (*e*) Describe any sensitivity analyses |  | NA |
| Results | | | | |
| Participants | 13* | (a) Report numbers of individuals at each stage of study—eg numbers potentially eligible, examined for eligibility, confirmed eligible, included in the study, completing follow-up, and analysed | Page 10 | Line 169-175.  Figure 2 |
|  |  | (b) Give reasons for non-participation at each stage |  | NA |
|  |  | (c) Consider use of a flow diagram |  | Figure 2 |
| Descriptive data | 14* | (a) Give characteristics of study participants (eg demographic, clinical, social) and information on exposures and potential confounders | Page 12-13 | Table 1 |
|  |  | (b) Indicate number of participants with missing data for each variable of interest | Page 9 | those with disease site not reported were excluded (line 170)  The HHC without reports of TPT outcome were categorized as ‘Outcome not recorded. (line 156) |
|  |  | (c) *Cohort study*—Summarise follow-up time (eg, average and total amount) |  | Not done. Since a lot of outcomes were not recorded, we are also not aware about the last day of follow up that these HHC received. |
| Outcome data | 15* | *Cohort study*—Report numbers of outcome events or summary measures over time | Page 10 | Line 177-184 |
|  |  | *Case-control study—*Report numbers in each exposure category, or summary measures of exposure |  |  |
|  |  | *Cross-sectional study—*Report numbers of outcome events or summary measures |  |  |
| Main results | 16 | (*a*) Give unadjusted estimates and, if applicable, confounder-adjusted estimates and their precision (eg, 95% confidence interval). Make clear which confounders were adjusted for and why they were included |  | NA |
|  |  | (*b*) Report category boundaries when continuous variables were categorized |  | NA |
|  |  | (*c*) If relevant, consider translating estimates of relative risk into absolute risk for a meaningful time period |  | NA |

Continued on next page

| Other analyses | 17 | Report other analyses done—eg analyses of subgroups and interactions, and sensitivity analyses | NA |  |
| --- | --- | --- | --- | --- |
| Discussion | | | | |
| Key results | 18 | Summarise key results with reference to study objectives | Page 13 | Line 209-213 |
| Limitations | 19 | Discuss limitations of the study, taking into account sources of potential bias or imprecision. Discuss both direction and magnitude of any potential bias | Page 16 | Line 265-273 |
| Interpretation | 20 | Give a cautious overall interpretation of results considering objectives, limitations, multiplicity of analyses, results from similar studies, and other relevant evidence | Page 12-15 |  |
| Generalisability | 21 | Discuss the generalisability (external validity) of the study results | NA |  |
| Other information | |  | | |
| Funding | 22 | Give the source of funding and the role of the funders for the present study and, if applicable, for the original study on which the present article is based |  | Has been mentioned as per journal guidelines |

*Give information separately for cases and controls in case-control studies and, if applicable, for exposed and unexposed groups in cohort and cross-sectional studies.

**Note:** An Explanation and Elaboration article discusses each checklist item and gives methodological background and published examples of transparent reporting. The STROBE checklist is best used in conjunction with this article (freely available on the Web sites of PLoS Medicine at http://www.plosmedicine.org/, Annals of Internal Medicine at http://www.annals.org/, and Epidemiology at http://www.epidem.com/). Information on the STROBE Initiative is available at www.strobe-statement.org.
