# Supplementary material for: Identifying missed opportunities in tuberculosis preventive treatment care cascade: Analysis of programme data from Maharashtra, India
Source: PLOS Glob Public Health. 2026 Jan 16;6(1):e0004630. doi: 10.1371/journal.pgph.0004630 (PMC12810897; doi:10.1371/journal.pgph.0004630)
Supplement: Attachment — Submitted filename: response to reviewers.docx [file pgph.0004630.s003.docx]

Manuscript No: PGPH-D-25-00926

**Title:** Identifying missed opportunities in tuberculosis preventive treatment care cascade: Analysis of programme data from Maharashtra, India

Dear Editor and reviewers,

First of all, I would like to thank you on behalf of all the authors for sparing your valuable time to go through the manuscript and give your comments. I am sure that this will help improve the overall quality of the manuscript. Please find the detailed responses to the comments in the below table. The line number mentioned here correspond to the line numbers in the clean version of the manuscript.

| **Sr. no.** | **Reviewer’s comments** | **Authors response** |
| --- | --- | --- |
| Reviewer #1 | | |
| 1. | Line 110: The authors refer to other TPT regimens, but do not describe them. These regimens will only appear in the results. | Thank you for the comment.  The other TPT regimens are introduced in the manuscript. line 114-118 |
| 2. | Line 115: PHC: this acronym needs to be translated | Thank you for the comment.  The acronym has been replaced with the full form. Line 123 |
| 3. | Line 141: Why did the authors consider as complete treatment those who died, were lost to follow-up, interrupted treatment due to toxicity and those without a final evaluation? I suggest excluding them from this category, showing the percentage found. | Thank you for the comment.  We did not include these categories in TPT completed but categorized them as TPT not completed. However, we understand there might be some ambiguity in the way the statement was articulated. We have modified it for better clarity.  Line 152-157 |
| 4. | Finally, in the Methodology and Results sections, I suggest including a figure including the “n” of each stage. The numbers presented are very confusing, causing the reader to have to go back to the original numbers to understand the cascade presented. | Thank you for the comment.  We had submitted a figure (now cited as figure 2) with the original submission for this showing the numbers for all the steps of the cascade. |
| Reviewer #2 | | |
|  | Abstract: |  |
| 1. | Use of commas in the reported numbers are inconsistent in use and placement. | Thank you for the comment.  We have corrected it at all places including the tables |
| 2. | Authors state that “household contacts of all people with pulmonary tuberculosis after ruling out active disease.” You then note that only 45% of HHCs were listed as eligible for TPT. Is that because others had active disease? Or because it was only prescribed in 45%? Would be helpful to understand who was diagnosed with active disease to remove from the cascade for the next step. | Thank you for the comment.  The national guidelines state that ‘all listed HHC should be screened for TB symptoms. Those having symptoms should be evaluated for TB infection as well as TB disease. Those having the disease should be put on DOTS regimen. Those having TBI should be initiated on TPT. However, in absence of TBI testing services, those unable to be evaluated for TBI should also be considered eligible and initiated on TPT after ruling out active TB disease.’ This part has been explained in the “specific setting” part of the manuscript.  Please note that of the 55% ineligible for TPT, only 799 were diagnosed with TB. This has been shown in Figure 2 submitted along with the initial submission. Since, the reasons for those labelled ineligible for TPT is not being recorded by the programme (in the mentioned data sources) and hence we are unable to comment on that. Also, the contact tracing register (from which this part of the analysis is done) contains aggregate data, based on which it is not possible to understand the eligible and non-eligible HHC at each step of the cascade. |
|  | Introduction: |  |
| 3. | “One recent strategy of NTEP is focusing on screening of TB and TPT provision in HHC (aged more than 6 years) of PwPTB.” What about children 5 and under? They are typically a group who are vulnerable and recommended TPT when exposed. | Thank you for the comment.  TPT for children less than 6 years is in place since several years in India. We understand that there might have been some ambiguity in way it was articulated. We have modified the statement for better clarity. Line 74-75 |
| 4. | It is noted that there are existing studies reporting challenges and losses in the care cascade already in districts of Maharashtra. How is this study different—includes the entire state of Maharashtra? Is it updated and including a newer time period? Were lessons learned from the previously identified challenges—processes, logistics, community engagement, acceptance—related to losses in the care cascade addressed since the last study? If so, should we expect to see improvement across the cascade? Please provide more clear rationale for why this study was conducted. | Thank you for the comment.  Yes, there is some evidence on challenges and losses in the care cascade in Maharashtra state based on a previous study (ref no 16). The study cited here was conducted in **research mode** in **selected few districts**. It was conducted at the time TPT was initiated and helped the government establish the implementation of the relevant services in the state.  The current study was conducted in **programme setting** and covers data for the **whole state of Maharashtra**. As this study is based on **secondary programme data**, there was no information related to the challenges in the data sources and hence we are unable to comment on the same.  We have modified a few lines to provide better clarity in this aspect. Line 80-84 |
|  | Methods |  |
| 5. | Study setting and specific TB epidemiology seem spread across the introduction and methods; would be better to consolidate. | Thank you for the comment.  We have modified the text based on the comment, as relevant for the study rationale. |
| 6. | It sounds like current strategy is to rule out TB disease, test (TST or IGRA) for TBI, and then provide TPT. However, if testing is not available, they are also provided with TPT. Can you please clarify this—is there a time period at which it is determined they need TPT despite no test available. Are there certain facilities that do not offer TST or IGRA? What is the variability of procedures/testing availability across the study area, as this will inform eligibility for TPT determination and potential delayed initiation? | Thank you for the comment.  There is no defined time period after which the eligibility has to be defined. The testing is to be provided by the public health system, but at the time corresponding to the data i.e in 2023, the infrastructure and capacity of the health system were limited to conduct these tests and the situation is gradually improving. For a vast majority of beneficiaries, in 2023, the situation would have been – test not available and they would have been initiated on TPT without testing for TB infection, at the earliest. |
| 7. | What are procedures/eligibility to determine who gets which regimen? Regimen type may differentially impact completion rates based on duration, side effect, etc. | Thank you for the comment.  A very valid point made regarding the effect of different regimens on completion rates.  For conditions which mandate a specific regimen (e.g. 1HP, 4R, 6lfx), it will be provided as per indications, but otherwise (e.g. for 3HP, 3RH, 6H) it is more of availability, i.e. the regiment which the state government endorses and makes available will be provided.  During 2023, the regimen was majorly 6H regimen in Maharashtra. Other regimens like 4R, 1HP and 6Lfx have specific indications. 3 HP/ 3RH although advocated, had limited availability. These indications have now been included in the manuscript (line no 114-118)  Since the treatment outcomes were not recorded for around 56% HHC, we did not carry out any further analysis to statistically compare the treatment outcomes across the regimens. |
| 8. | The description of who is responsible for what (lines 111-117) are unclear. Also, what type of capacity building and supportive supervision and support for treatment adherence are provided? Are these actually adhered to? | Thank you for the comment. We have tried to simplify the statement mentioning the exact cadres who are responsible for the activities. (line 118-125)  Capacity building here included training the providers in listing contacts, symptomatic screening/ evaluation and counselling. Supportive supervision means that the STS would try to understand the performance and activities of the workers and provide handholding/ suggest strategies for effectively carrying out the required activities. He is also supposed to visit the beneficiaries, counsel them and provide any support if needed.  There may be gaps in the actual implementation of these activities. However, since this manuscript focusses on secondary data analysis, it is out of our scope to comment on this aspect in this manuscript. |
| 9. | When contact tracing is done, is it just household contact or close contacts? Please provide the definition. | Thank you for the comment.  The national guidelines for programmatic management of tuberculosis preventive treatment mentions that contact tracing and enumeration of target population should be done on a weekly basis. This along with the definition of the contacts is now added in the manuscript. (line 106-109) |
| 10. | Did the index patient need to have bacteriologic confirmation of disease in order for contact tracing to be done? | Thank you for the comment.  No, contact tracing is to be done for all persons getting diagnosed with pulmonary TB (clinically diagnosed or microbiologically confirmed). |
| 11. | Please clarify who was included. Did the diagnosis of pulmonary TB for the index patient need to take place from Jan-Dec 2023? If so, it takes time for contact tracing, testing, screening, treatment initiation, and completion. Were contacts followed for a longer period of time to make sure they had time to complete? | Thank you for the comment.  Yes, as has been mentioned in the study population, all PwPTB who are diagnosed between Jan-Dec 2023 were included.  Once a patient is diagnosed, his/her details are notified in the notification register of *Ni-kshay* along with the date of diagnosis*.* The information of those with notification dates within the mentioned study duration was extracted for this study and analysed.  For the longest regimen (6H) to complete, it would take around 239 days which is approximately 8 months (the maximum allowable time). As further mentioned in the manuscript (line 150-151), the data was downloaded in Nov 2024 (last week). This is approximately 11 months after the last patient would have been notified (on 31^st^ Dec 2023). Hence, there was sufficient time to make sure treatment was completed for those who intended to do so and also allowing for some time lag for data entry into the *Ni-kshay*. |
| 12. | Does ‘not evaluated’ indicate a response was missing or that not enough time had passed and they could still be on treatment at the time of data download? | Thank you for the comment.  Not evaluated is a specific outcome defined under the national programme which should be categorized in instances (quoting from the guidelines) “Such as records lost, transfer to another health facility without record of TPT completion.” – ref no 12  The mentioned outcomes have been given by the programme staff as per the guidelines. |
|  | Results: |  |
| 13. | Same comment as earlier about commas in numeric values. | Thank you for the comment.  We have corrected it at all places including the tables |
| 14. | Please clarify the exclusionary criteria of ‘outliers with respect to number of HHC’. How was this defined? Please include in methods. | Thank you for the comment.  The HHC with total number of HHC >20 or number of HHC (aged ≤ 6 years) >10 were considered as outliers.  This has now been included in methods (line 148-149) |
| 15. | Why were only 1,85,502 HHC eligible for TPT? Did others have active disease? Shouldn’t everyone except those with active disease be eligible? | Thank you for the comment.  There are two models for TPT. The first – **“test and treat” model,** according to which, all contacts with TB infection and without active TB are eligible for TPT. The second – “**treat all”** model according to which all individuals without active TB are eligible for TPT. India follows the test and treat model.  These number are derived from the “contact tracing register” which has aggregate data. Further, the register captures numbers evaluated for TB infection but does not capture how many had TB infection. It also captures numbers diagnosed with active TB disease. Hence, it is difficult to comment on how many were positive for TB infection. We have mentioned the number of those diagnosed with TB disease in figure 2 submitted with the manuscript. |
| 16. | It seems like Line 161 should come after Lines 162-166. | Thank you for the comment.  The sentence has been removed as it was already coming later in the paragraph. |
| 17. | If only 3% of HHCs did not complete TPT, what were the reasons, and what happened to the rest if only 41% successfully completed? Later it says that treatment outcomes for 56% of the contacts was not recorded. Is this because there was not time to complete it before data were recorded or collected? I think this is a major issue that is not addressed or defined appropriately in the manuscript. | Thank you for the comment.  The *Ni-kshay* registers do not capture the reasons for loss to follow up/ non-completion of treatment. Hence, it would be difficult for us to tell the exact reasons. However, a break up of different outcomes (from these 3%) have been mentioned in the care cascade figure (figure 2) submitted with the manuscript earlier.  Out of the total (100%) of those who were initiated on TPT, the break-up of different outcomes was (outcome not recorded – 56%, TPT completed – 41%, TPT not completed – 3%). For better understanding we have added another row in the table with overall figures and modified the respective statements in the text. (line 187-189)  Regarding the time to completion, requesting you to kindly refer to the response for comment no. 11 earlier. |
| 18. | Results talk about stratification by private vs public sector, but there was no information about how services or the patients who attend these facilities differ. | Thank you for the comment.  A very valid point again regarding the difference in services between private and public sector.  The type of sector described here i.e. private and public is for the Index TB patients and not HHC. However, during the said duration of the study, the availability of TPT related services was very limited in the private settings. During that time period the HHC were to be referred to the programme settings for services related to TPT.  Further, as there was limited information available on the type of service in different settings in the data sources, we were unable to do any further analysis in that aspect and report. |
|  | Editorial comments |  |
| 1. | Although the findings are not novel, we feel that there is potential for publication if the issues raised by the reviewers and by myself are addressed. | Thank you for the kind comment.  We have tried our best to address all the issues raised. Should there be any other issues, we shall be happy to address them as well. |
| 2. | Clearly, the national (or state) guidelines need to be clarified for the audience to understand your results, as pointed out by the reviewers. Maybe a figure on the algorithm for HHC management would help the reader to understand your numbers. | Thank you for the comment.  We have added a figure (Figure 1) from the national guidelines showing the care cascade after the specific settings. |
| 3. | It is not clear why so many people were considered not eligible, and sentences such as lines 173-174 (41% completed and 3% did not complete) are not clear. | Thank you for the comment.  It was indeed a point of concern for us too. Since this particular information was taken from the contact tracing register, which is an aggregate register, it is difficult to understand how many were eligible/not eligible at each step of the cascade (mentioned in limitations too).  Requesting you to kindly refer to response to comment no. 17 of reviewer 2 in this document. |
| 4. | In this same setting subheading, please explain what regimens are available and how are they selected. Healthcare worker or patient's choice? Are there specific indications for different regimens? If so, this might impact on treatment outcomes and should be considered in the interpretation of findings. | Thank you for the comment.  In accordance with this comment and also of one of the reviewers, we have now included the indications for different regimens in the manuscript (line no 114-118).  For conditions which mandate a specific regimen (e.g. 1HP, 4R, 6lfx), it will be provided as per indications, but otherwise (e.g. for 3HP, 3RH, 6H) it is more of availability, i.e. the regimen which the state government endorses and makes available will be provided.  During 2023, the regimen was majorly 6H in Maharashtra. Other regimens like 4R, 1HP and 6Lfx have specific indications. These indications have now been included in the manuscript. 3 HP/ 3RH although advocated, had limited availability during the mentioned study duration. |
| 5. | Please justify your choices of outcomes for comparison against completed, which are so different and have very different meanings. A complementary analysis of completed of TPT completion versus biological outcomes could bring light to your findings and discussion. | Thank you for the comment.  The outcomes as has been mentioned in the manuscript are as per the programme guidelines. We have operationally defined the merged categories for our analysis. (line 153-157)  For the purpose of this study, we operationally classified all treatment outcomes other than ‘TPT completed’ (died, lost to follow-up, treatment failed, TPT discontinuation due to toxicity, and not evaluated) as ‘TPT not completed’. The HHCs without reports of TPT outcome were categorized as ‘Outcome not recorded.  Further, as the treatment outcomes for 56% of the contacts was not recorded, we have not carried out any further analysis. |
| 6. | Your discussion needs thorough revision. It should help the reader to understand the meaning of your findings. Do not repeat (twice) the results in the discussion, just summarize them in the first paragraph and then interpret them under the light of the literature and of the limitations of the study. | Thank you for the comment.  We have tried to modify the discussion in line with the suggestion. |
| 7. | Please include the very high prevalence of TB found in contacts, this has very relevant public health implications on recommendations for contact tracing as well. Think on what could explain your results. In this matter, please cite the possible bias of non-completion results (or other outcomes) among those who were not reported. Also mention the nature of this secondary data observational study (the design) as a limitation. | Thank you for the comment.  We have included this high prevalence of TB infection in contacts in the discussion (line 224-225) and also tried to modify some parts of discussion in an attempt to try to explain possible explanations for the results.  We have included the suggested limitations in the manuscript (line 265-273) |
| 8. | Your conclusions, except form the first sentence, are not conclusions from your data. Please change this subheading to Recommendations or Final considerations. | Thank you for the comment.  We have changed the subheading as suggested. |
| 9. | A STROBE check list would be helpful, please attach one in your resubmission. | Thank you for the comment.  We have prepared and submitted the STROBE checklist as suggested. |
| 10. | Please update your definition of TBI (Coussens et al) in the Introduction. | Thank you for the comment.  We have updated the definition as per the suggestion. (Line 61-63) |
| 11. | Please update your references and rather use systematic reviews instead of single studies. Global reports are more reliable than European data to discuss your findings. | Thank you for the comment.  We have included a few more systematic reviews in the discussion part. We have used some single studies from India purposively to bring in the local context. |
| 12. | When revising, please amend the numbers to the occidental annotation. We understand the authors used the Indian numeric system, but this is an international journal, read by a global audience. | Thank you for the comment.  We have corrected it at all places including the tables. |
