# Supplementary material for: Identifying missed opportunities in tuberculosis preventive treatment care cascade: Analysis of programme data from Maharashtra, India
Source: PLOS Glob Public Health. 2026 Jan 16;6(1):e0004630. doi: 10.1371/journal.pgph.0004630 (PMC12810897; doi:10.1371/journal.pgph.0004630)
Supplement: Attachment — Submitted filename: response to reviewers_r2.docx [file pgph.0004630.s004.docx]

**Manuscript No: PGPH-D-25-00926**

**Title:** Identifying missed opportunities in tuberculosis preventive treatment care cascade: Analysis of programme data from Maharashtra, India

Dear Editor and reviewers,

First of all, I would like to thank you on behalf of all the authors for sparing your valuable time to go through the manuscript and give your comments. I am sure that this will help improve the overall quality of the manuscript. Please find the detailed responses to the comments in the below table. The line number mentioned here correspond to the line numbers in the clean version of the manuscript.

| **Sr. no.** | **Reviewer’s comments** | **Authors response** |
| --- | --- | --- |
| Reviewer #1 | | |
| 1. | Line 129 – Those who did not receive skin testing or other tests: Why? Was there no availability at the time of screening? I suggest adding clarification. The authors should be clearer about how HHC were extracted from the reporting system, as well as clarify how the TPT regimen were chosen for HHC (only availability at the time of treatment initiation or was there another criterion?) | Thank you for the comment.  The said statement is paraphrased from the national guidelines. In the context of this study, for the time period corresponding to which the data has been analysed, since the program was scaling up, the test availability was limited and that would have been the reason for no test in majority of the cases.  We have added text to provide some more clarity in lines 110-122.  We have also added a statement “TPT was provided to the beneficiaries as per the indications and availability” in line 129-130 to clarify how TPT regimen was given to a particular HHC |
| 2. | Figure 1:   - Needs a title. - Does the target population refer to contacts? This needs to be made clear. - What do the authors call presumptive TB? Have these HHC presented some symptoms? - Evaluate for TPT means eligibility criteria? | Thank you for the comment.  We have modified the figure for better clarity.  The journal guidelines tell that we should not include the title in the figure but should write the title as a text in the manuscript while submission. We have written the title in the manuscript at appropriate place (line 138-139). |
| 3. | Figure 2:   - Needs a title - The numbers in Figure 2 were not changed, so they need to be corrected to align with the text. | Thank you for pointing this out.  There were a few mistakes in the numbers in both the text and figure. We have corrected that and uploaded a new figure. (lines 189, 201-202, table 1)  Title has been written in line 198-199 |
| 4. | Line 266 – How were contacts diagnosed with active TB (X-ray? Physical examination? Bacteriological examination?) | Thank you for the comment.  We have added the following text to clarify this in the methods section. (line 110-114)  “Symptomatic HHC are evaluated for presence of active TB disease using chest X-ray and bacteriological examination of sputum, and only those free of active disease are considered for TBI evaluation. Among the asymptomatic contacts aged 6 years or more, active TB disease is ruled out using chest X-ray” |
| Reviewer #3 | | |
| 1. | One important issue requiring more attention is the criteria used to determine eligibility for TPT, especially regarding contacts who were not adequately assessed for symptoms or whose symptom assessment status was unclear. Currently, it is not entirely clear how contacts who were either symptomatic and not thoroughly evaluated or asymptomatic and did not have confirmatory tests (IGRA or TST) were classified as eligible for TPT. | Thank you for the comment.  We have tried to make it more clear by modifying some sentences from the methodology. Lines 110-122  Further, as all information related to eligibility for TPT is coming from contact tracing register, which has aggregate data, it is not possible to understand who underwent which test and what was the result. However, as per anecdotal understanding, information about TB evaluation is entered in the system for only those who are symptomatic. |
| 2. | Methods Section:  It would be helpful to include a brief but explicit description of the criteria used for TPT eligibility, particularly for contacts unable to complete TBI testing. For instance, consider specifying: “Household contacts unable to undergo IGRA or TST testing were considered eligible for TPT if they were asymptomatic upon initial clinical screening performed by trained health professionals, consistent with national program guidelines.” | Thank you for the comment.  As per the suggestion, we have added the following statement in Line 119-121  “HHC unable to undergo TBI testing due to any of these reasons are also considered eligible for TPT if they are free of active TB disease, consistent with national guidelines” |
| 3. | Indicate the number or proportion of contacts considered eligible based solely on symptom screening, without confirmatory IGRA or TST tests. For example: “Among eligible contacts, XX% were included based exclusively on clinical screening (absence of symptoms), without IGRA or TST testing.” | Thank you for the comment.  Unfortunately, we are unable to comment on the proportion of HHC considered eligible solely on the basis of (absence of) clinical symptoms. This information ideally, should be available in the contact tracing register but currently the register captures information only about TB disease evaluation and not TBI evaluation. There is a possibility of getting this information from the TPT register, where the “basis of diagnosis” needs to be entered. However, despite our best-efforts during data cleaning, we could not derive any meaningful numbers, as for over 80% HHC initiated on TPT, the entry mentioned “others” which does not make much sense from a research point of view. We have modified the limitations to reflect the same and added the following statement in lines 288-291.  “Although, the contact tracing register captures numbers evaluated for and diagnosed with TB disease, it does not capture the number of HHC evaluated for TBI or those diagnosed with TBI, hence, we are unable to assess the gaps in TBI evaluation of HHC under the national programme.” |
| 4. | Discuss the implications of determining TPT eligibility without confirmatory diagnostic testing, emphasizing potential limitations such as overtreatment or the risk of missing subclinical active TB cases. | Thank you for the comment.  We have added the implications as suggested in line numbers 252-258 |
| 5. | Additionally, the results would benefit from explicitly reporting percentages or numbers at each stage of the diagnostic process. | Thank you for the comment.  We have added a few more percentages explicitly in the results (line 191-194) which were not mentioned earlier. However, one limitation as mentioned earlier is that the data registers specified in this study do not capture information related to how many were tested for TBI and how many had TBI. Hence, we regret to say that we are unable to comment on that aspect from. |

In addition to these changes, we have also modified the abstract a bit.
