# Supplementary material for: Identifying missed opportunities in tuberculosis preventive treatment care cascade: Analysis of programme data from Maharashtra, India
Source: PLOS Glob Public Health. 2026 Jan 16;6(1):e0004630. doi: 10.1371/journal.pgph.0004630 (PMC12810897; doi:10.1371/journal.pgph.0004630)
Supplement: Attachment — Submitted filename: response to reviewers_r3.docx [file pgph.0004630.s005.docx]

**Manuscript No: PGPH-D-25-00926**

**Title:** Identifying missed opportunities in tuberculosis preventive treatment care cascade: Analysis of programme data from Maharashtra, India

Dear Editor and reviewers,

First of all, I would like to thank you on behalf of all the authors for sparing your valuable time to go through the manuscript and give your comments. I am sure that this will help improve the overall quality of the manuscript. Please find the detailed responses to the comments in the below table. The line number mentioned here correspond to the line numbers in the clean version of the manuscript.

| **Sr. no.** | **Reviewer’s comments** | **Authors response** |
| --- | --- | --- |
| Reviewer #1 | | |
| 1. | Line 184 - 84% of 133,167 is 111,860, not 112,034 (contact tracing) | Thank you for the comment. Actually, we had rounded of the percentage. The actual percentage is 84.13%, which we have now revised. Line 185 |
| 2. | Line 207 - TPT completion was similar for all age groups and for both the genders. I suggest “all genders” because the authors list three genders in the table | Thank you for the comment. We have modified the statement as per your kind suggestion. Line 207 |
| Reviewer #4 | | |
| 1. | Title: It is accurate for the work done, although I have some reservation with the word ‘analysis’, as discussed below. | Thank you for the comment.  Although we agree that analysis would mean that some comparison is done between 2 or more groups, with due respect to the reviewers, we wish to keep it in the title as it is since analysis is also needed for presenting descriptive results and often such results are presented using the term ‘descriptive analysis’. However, if the reviewers and editors still feel otherwise, we are open to changing this. |
| 2. | Study Design: The authors have described study design as retrospective cohort study, as well as used the word ‘Analysis of programme data’ in their title. But it actually a descriptive record based study. In the standard epidemiological study classification, cohort study is classified under analytical studies and done to testing of hypothesis with the use of some effect size to measure the significance association. Like OR, RR, and performing parametric and or non paramatric tests. The present manuscript have not performed any testing of hypothesis, but only description of routinely collected data. It seem to fit more as described record beased study than a retrospective cohort study. | Thank you for the comment.  The reviewers have aptly stated that cohort is classified under analytical studies.  However, a cohort study can be both descriptive and analytical depending on description of incident outcomes from one group (descriptive) and comparison of incident outcomes between two groups (analytical).  There are published examples of cohort studies where the results are descriptive. https://pubmed.ncbi.nlm.nih.gov/37496608/ |
| 3. | Study settings: Since Maharashtra is a large state and TPT is being delivered utilizing various mechanisms - Treat only, Test and treat, Montox, CyTB, public–private partnership, along with as a part of routine service. This need to be well documented and even observed across TPT treatment outcome in the table 1 if data is available. | Thank you for the comment.  This is indeed true and there might have been variations in different regions of Maharashtra. However, unfortunately, the data source registers do not capture information on the mechanism (Treat only, Test and treat).  The contact tracing register being an aggregate data, does not capture these information for each patient. It only captures the number of contacts identified, numbers tested for TB, numbers diagnosed with TB, numbers eligible for TPT and numbers initiated on TPT.  Regarding the type of test used, although the basis of diagnosis (tests for TB infection) needs to be entered for every household contact initiated on TPT, it was not done properly at the time corresponding to the study period. Despite our best-efforts during data cleaning, we could not derive any meaningful numbers, as for over 80% HHC initiated on TPT, the entry mentioned “others” which does not make much sense from a research point of view.  Regarding Public/ private, the outcomes according to the current health facility (whether public or private) has been presented in table 1. |
| 4. | Results: Well presented. Can we add OR in the tables? | Thank you for the comment.  We initially thought about inferential statistics. However, due to a large amount of missing data pertaining to treatment outcomes and losses from the cascade which would have influenced the analysis and the results would not have been very meaningful, we refrained from doing it. |
